# Breastfeeding support and barriers to women with gestational diabetes mellitus: a nationwide cross-sectional survey of hospitals in Japan

**DOI:** 10.1186/s12884-021-04032-9

**Published:** 2021-08-13

**Authors:** Mayumi Matsunaga, Yaeko Kataoka, Yumiko Igarashi, Toshiko Fukui, Masumi Imura, Shigeko Horiuchi

**Affiliations:** 1 St. Luke’s International University- Graduate School, Women’s Health and Midwifery, Tokyo, Japan; 2 Midwifery Policy Committee, Japan Academy of Midwifery, Tokyo, Japan; 3 Japanese Nursing Association, Executive Board, Tokyo, Japan; 4 Global Health Care and Midwifery, Graduate School of Nursing, Japanese Red Cross College of Nursing, Tokyo, Japan

**Keywords:** Gestational diabetes mellitus, Breastfeeding support, Barriers, Nurses, Midwives

## Abstract

**Background:**

Despite the benefits of breastfeeding for women with Gestational Diabetes Mellitus (GDM) and their infants, breastfeeding is less likely to be performed by this group. This study aimed to examine the current levels of implementation of breastfeeding support to women with GDM in Japan and to clarify barriers to promoting breastfeeding among this population.

**Methods:**

A 25-item questionnaire was developed by the authors to investigate the current levels of implementation of breastfeeding support for women with GDM provided in hospitals, and to explore barriers for promoting breastfeeding among these women. The questionnaire was sent to all 1046 hospitals facilitating childbirth in Japan. Descriptive statistics were used to analyze the quantitative data, and content analysis was used to analyze qualitative data from the open-ended questions.

**Results:**

All 296 respondents were included in this study. Regarding breastfeeding support, 95.2% of the respondents provided general information on breastfeeding to GDM women during antenatal midwife consultations. However, the benefits of breastfeeding for preventing type 2 diabetes were addressed by only 48.0%. Likewise, although follow-up services (e.g., telephone support or breastfeeding consultations) were conducted in 88.9% of hospitals, only 50.7% of hospitals informed women that breastfeeding decreases the risk of developing type 2 diabetes after GDM. Regarding barriers, seven categories and 20 subcategories about promoting breastfeeding for women with GDM were extracted and abstracted into the following three themes: *Barriers associated with mother and infant, Barriers associated with health professionals,* and *Organizational barriers.*

**Conclusions:**

In Japan, most hospitals that responded provided general breastfeeding support from the antenatal to postpartum periods. However, the benefits of breastfeeding in terms of preventing the incidence of type 2 diabetes following GDM were insufficiently communicated to women with GDM. Furthermore, there were numerous barriers to promoting breastfeeding among women with GDM.

## Background

Gestational diabetes mellitus (GDM) is a complication of pregnancy characterized by maternal hyperglycemia. Recently, the prevalence of women diagnosed with GDM has been increasing worldwide, with an incidence rate of approximately 5.8-12.9% [1]. GDM is associated with significant metabolic health issues for the mother and infant, not only during pregnancy, but also long term. Infants born to diabetic mothers are at increased risk of obesity compared with those born to healthy mothers [2]. Women diagnosed with GDM have a seven times higher risk of developing type 2 diabetes mellitus than women who have normal glucose levels during their pregnancy [3]. The estimated risks for type 2 diabetes mellitus after GDM were from 19.7% at 10 years to 39.0% at 30 years [4]. Owing to these long-term implications for metabolic health, effective management of GDM is one of the most urgent health challenges in this field.

Moderate evidence has attributed long-term benefits to breastfeeding for both mothers with GDM and their children. First, regarding the benefit to children, breastfeeding was associated with a reduction in the risk of both obesity [5] and the incidence of type 2 diabetes [6].Second, regarding the potential for breastfeeding to reduce the incidence of type 2 diabetes mellitus among women with a GDM history, Tanase-Nakao et al. [7] discovered, in their systematic review, that longer and exclusive lactation might be beneficial for reducing type 2 diabetes mellitus prevention among women with previous GMD. Furthermore, Yasuhi et al. [8] found that the development of abnormal glucose tolerance during the first postpartum year could be prevented by high-intensity breastfeeding for longer than 6 months by improving Japanese women’s insulin resistance independent of postpartum weight change and obesity.

Despite such potential benefits of breastfeeding for women with GDM and their infants, a systematic review [9] that included 16 studies concluded by noting that breastfeeding is less likely to be initiated or continued by women with GDM than by women with healthy pregnancies. Likewise, according to a study conducted in Japan [10], the breastfeeding rate among women with GDM is lower than that of women without GDM. There are many factors that influence the initiation and continuation of women’s breastfeeding [11]. However, according to Rollins et al. [12], the health system and services are among the main factors associated with the status of breastfeeding. Thus, it is assumed that the reason for the lower rate of breastfeeding among women with GDM might be related to their insufficient or inappropriate support by health professionals.

In Japan, generally pregnant women with GDM are considered as high-risk and their care is managed within a hospital setting with nurses and midwives providing a significant amount of breastfeeding support. Yet, little is known about their current service provision of breastfeeding support. This study aimed to examine the current status of breastfeeding support provided by nurses and midwives for women with GDM in Japanese hospitals, and to identify the barriers to promoting breastfeeding among this population. It was part of a larger study examining interprofessional management practices for women with GDM.

## Methods

### Study design

A nationwide cross-sectional survey was conducted to examine breastfeeding support performed by nurses and midwives for women with GDM in hospital settings in Japan. The methodology conformed to the Strengthening the Reporting of Observational Studies in Epidemiology (STROBE) guidelines [13] and the Standards for Reporting Qualitative Research (SRQR) [14].

### Participants and procedure

The purpose of this study was to investigate the current practices within hospitals in Japan for promoting breastfeeding among women with GDM. Therefore, a questionnaire was mailed by post to all 1046 hospitals in Japan, as of 2019, that provided antenatal, intrapartum and postpartum services for women with GDM. The questionnaire required a senior midwife or nurse, who was familiar with the hospital’s practices and services for women with GDM, to answer the questionnaire. In order to increase the response rate, 2 follow-up reminders were sent to all hospitals. Data were collected anonymously between March 2019 to September, 2019.

### Ethical considerations

The Ethics Board of St. Luke’s International University provided ethical approval.

### Measures (questionnaire)

To the best of our knowledge, there are no exiting tools available to assess the service provision of breastfeeding support for women with GDM. Therefore, the authors developed a 25-item questionnaire to investigate the current levels of implementation of breastfeeding support for women with GDM provided in hospitals, and to explore barriers for promoting breastfeeding among these women. This questionnaire included 7 items related to the demographics of individual participants, and the demographic characteristics of their hospitals. There were 15 questions that investigated the level of implementation of breastfeeding support for women with GDM in the hospitals and were scored using a 4-point Likert scale (i.e., not at all, very little, to some extent, to a great extent). First, these 15 questions were developed based on *the Ten Steps to Successful Breastfeeding* [15], which was jointly published by the World Health Organization (WHO) and the United Nations International Children’s Emergency Fund (UNICEF) and was defined as *basic support* in the present study. Second, the questions were modified if the support conditions were to be tailored specifically for women with GDM. Furthermore, some questions regarding specific forms of educational support for women with GDM were added to the questionnaire. These specific forms of support for the special needs of women with GDM were defined as *specific support* in the present study. All 15 questions are facilitators of breastfeeding for women with GDM. To tap into barriers to breastfeeding during the antenatal, intrapartum, and postpartum periods the questionnaire also included three open-ended questions about what participants thought were barriers. A senior International Board-Certified Lactation Consultant (IBCLC) checked the questionnaire for face validity, followed by a pilot test with five clinical midwives who also provided face validity.

### Data analysis

For quantitative data collected from closed-ended questions, and the 4-point Likert scales, statistical analyses, including descriptive statistics (frequency, percentage, mean, and SD) were performed, using SPSS version 27. For qualitative data collected by open-ended questions, content analysis [16] was performed using NVivo software ver. 12. The aim of content analysis is to analyze nursing sensitive phenomena or unknown phenomenon [17], which made it a good fit with our research questions. The raw data were inductively coded and categorized to generate overarching themes. To ensure rigor in the qualitative data analysis, two authors (MM and YI) individually conducted coding and categorizing. When disagreements arose over the analysis, it was resolved by discussion with other authors (YK and SH) until consensus was reached. All researchers were midwives and that may have influenced the research results and their interpretation. A Japanese linguist and a native English researcher supervised the language accuracy of the categories.

## Results

A total of 308 hospitals responded (29.3%) in this study. Of those, 12 hospitals were excluded as they did not provide care service to women with GDM. Table 1 presents the demographic characteristics of the respondents and hospitals. The breakdown of the respondents’ profession was midwives (89.2%) and nurses (10.1%). The respondents’ positions were ward manager (47.3%), assistant ward manager (9.8%), chief (15.9%), and others (25.0%). The average total years in the profession was 23.31 years (SD 7.77) (median: 23.00 years, range:1-50 years), and the average years in obstetric was 18.81 years (SD 8.59) (median:19.00 years, range: 0-38).

**Table 1:** Demographic characteristics of the responding hospitals (*N* = 296)

	Number of participants (n)	Proportion of participants (%)
Hospital classification		
Tertiary perinatal hospital	47	15.9
Secondary perinatal hospital	93	31.4
Primary perinatal hospital	152	51.4
No response	4	1.4
Number of births per year		
< 500	166	56.1
500-1000	89	30.1
1001-1500	18	6.1
1501<	12	4.0
No response	11	3.7
Maternity service		
Midwifery outpatient services		
Yes	207	69.9
No	87	29.4
No response	2	0.7
Alongside midwifery unit		
Yes	34	11.5
No	260	87.8
No response	2	0.7
Breastfeeding outpatient consultation		
Yes	241	81.4
No	53	17.9
No response	2	0.7
Two weeks postnatal check-up		
Yes	205	69.3
No	89	30.0
No response	2	0.7
One-month postnatal check-ups		
Yes	278	93.9
No	16	5.4
No response	2	0.7

Regarding hospital classification, the hospitals were divided into primary perinatal hospital (51.4%), secondary perinatal hospital (31.4%) and tertiary perinatal hospital (15.9%). While most of the hospitals were located in the Kanto region (includes the greater Tokyo area), which accounted for 32.4% of the total, hospitals in all areas in Japan responded. Of these hospitals, most hospitals (86.2%) had 1000 deliveries a year or less. For maternity services, a majority of the hospitals provided one-month check-ups (93.9%), breastfeeding outpatient consultation (81.4%), and a relatively large number of hospitals provided midwifery outpatient service (69.9%). However, only a few hospitals provided ‘alongside-midwifery’ units (11.5%).

### Breastfeeding support

**Fig. 1:**
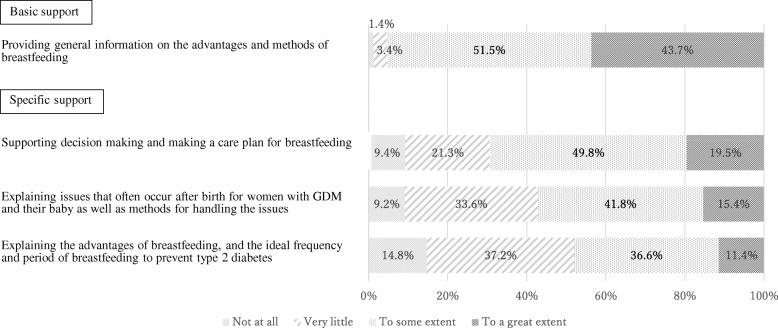
Levels of breastfeeding support provided during the antenatal period (*N =* 296)

**Fig. 2:**
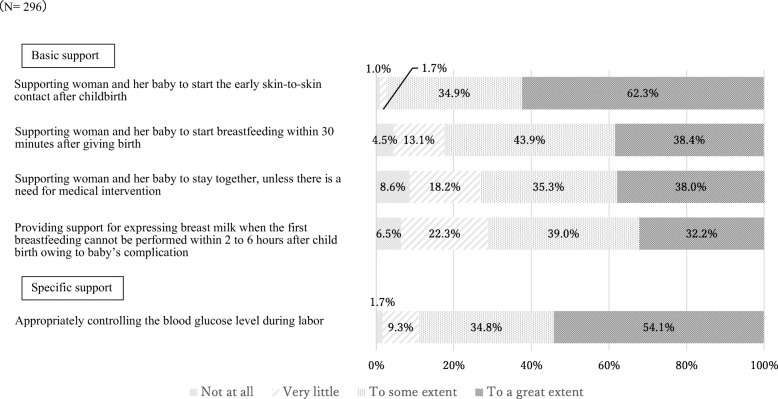
Levels of breastfeeding support provided during labor and right after childbirth (*N =* 296)

**Fig. 3:**
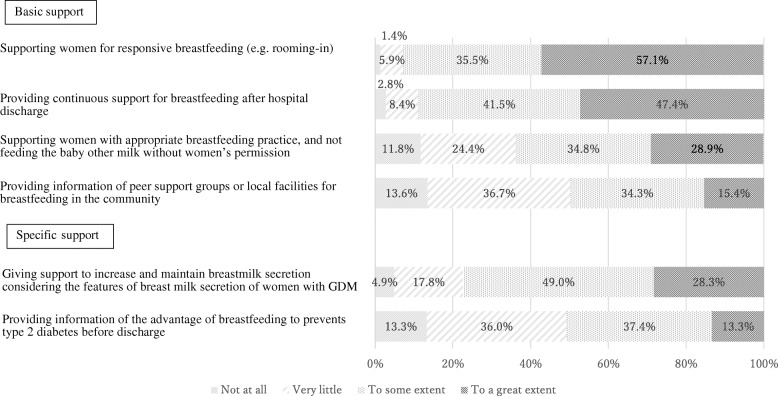
Levels of breastfeeding support provided during the postpartum period (*N* = 296)

The levels of breastfeeding support for women with GDM provided by nurses and midwives are shown in Figs. 1, 2 and 3. Respondents were asked about the level of support they provided to mothers with GDM during the antenatal, labor and immediately after, and postpartum periods.

### Antenatal period

Figure 1 presents the levels of breastfeeding support provided during the antenatal period. A majority of the hospitals (95.2%) provided basic support in the form of general information on the advantages and methods of breastfeeding. However, considerably fewer hospitals provided the specific support such as explaining the issues that often occur after birth for women with GDM and their baby as well as methods for handling the issues (57.2%) or the advantage of breastfeeding in preventing type 2 diabetes mellitus (48%). In particular, the number of hospitals that provided such specific support “to a great extent” was extremely small (range 10 - 20%).

### During labor and immediately following childbirth

Figure 2 presents the levels of breastfeeding support provided during labor and right after childbirth. Overall, comparing the support between the antenatal and postnatal periods, the support during labor and immediately after childbirth was relatively well provided. All items were performed in more than 70% of hospitals “to a great extent” and “to some extent”. In particular, a majority of hospitals (97.2%) provided support for early skin-to-skin contact, with 62.3% of these hospitals providing support “to a great extent”. Early skin-to-skin contact was the most well implemented support in most hospitals from among all the support items from pregnancy to postpartum.

### Postpartum period

Figure 3 presents the levels of breastfeeding support provided during the postpartum period. Regarding basic support during the postpartum period, a majority of hospitals provided support for ‘responsive breastfeeding’ (92.6%) and continuous breastfeeding support following discharge (e.g., telephone support or breastfeeding consultations) (88.9%). However, only about half of the hospitals (49.7%) reported providing information about local peer support groups and breastfeeding facilities. Regarding specific support during the postpartum period, similar to the antenatal period, only about half hospital (50.7%) provided information on the benefit of breastfeeding for the prevention of type 2 diabetes mellitus.

### Barriers to promote breastfeeding among women with GDM

There were 170 (55%) participants who answered the open-ended questions about what they thought were barriers to promote breastfeeding for women with GDM. Content analysis of this qualitative data regarding barriers led to the identification of 7 categories and 20 subcategories, which were linked to the following three themes: Barriers associated with mother and infant, Barriers associated with health professionals, and Organizational barriers. The details of the results are described below. The results are shown by category in italics and in single quotation marks by subcategory. Codes that emerged from the raw data, and abstracted into subcategories, are presented in narrative form. Table 2 presents the barriers to breastfeeding.

**Table 2:** Perceived barriers by nurse and midwives to promote breastfeeding (*n* = 170)

Theme	Category	Sub-category
Barriers associated with mother and infant	Medical interventions for infant	Separation of mother and infant following delivery due to admission of infant to GCU/NICU
		Supplementation of artificial milk for infant due to hypoglycemia
	Difficulties with breastfeeding due to mother’s condition	Physiological and physical characteristics among woman with GDM affecting breastfeeding
		Low intention of mothers and family to breastfeed
Barriers associated with health professionals	Issues around breastfeeding support for women with GDM at individual level	Low awareness among health professionals about GDM
		Lack of knowledge and skills among health professionals regarding GDM and breastfeeding support
		Lack of basic breastfeeding support for women with or without GDM
		Lack of additional support for breastfeeding among women with GDM
		Lack of health guidance on the effects of breastfeeding to prevent type 2 diabetes mellitus
	Issues around collaboration within professions and between different departments and professions	Differences in competence and approach to GDM and breastfeeding among nurse and midwives
		Lack of coordination among different health professionals and departments
		Differing approaches among health professionals to breastfeeding for women with GDM
Organizational barriers	Lack of resources for breastfeeding support	Lack of manpower
		Lack of time and opportunity to provide health guidance for breastfeeding
		Difficulty in securing time for staff training
	In-hospital support systems and policies that hinder the promotion of breastfeeding	Hospital management systems that make breastfeeding support difficult
		Hospital policies restricting early skin to skin contact, early initiation of lactation, and rooming-in
		Routine artificial milk supplements to infants
	Lack of continuous interprofessional support system	Lack of support following discharge
		Difficulties in collaborating with other facilities

### Barriers associated with mother and infant

The barriers included in this theme consisted of two categories: *Difficulties with breastfeeding related to medical interventions for infant* and *Difficulties with breastfeeding due to mother’s condition*.

*Difficulties with breastfeeding related to medical interventions for infant* included two sub-categories. The first subcategory was, ‘Separation of mother and infant following delivery due to admission of infant to Growing Care Unit (GCU) or Neonatal Intensive Care Unit (NICU)’. Respondents stated, for example, that if the baby had hypoglycemia and was admitted to the NICU/GCU (e.g., entering an incubator and having an IV drip), the mother would not be able to breastfeed unless the baby left the incubator. The second subcategory was ‘Supplementation of artificial milk for infant due to hypoglycemia’. A respondent explained that babies were often hypoglycemic after delivery and their first breastfeeding was likely to be formula milk rather than breast milk.

*Difficulties with breastfeeding due to mother’s condition* consisted of two subcategories: ‘Physiological and physical characteristics among woman with GDM affecting breastfeeding’ and ‘Low intention of mothers and family to breastfeed’. Regarding the former category, respondents stated that there were situations where it was difficult to promote breastfeeding in terms of the mother’s physical and/or mental condition. For example, respondents stated that there were many women with breast sizes and nipple shape that created difficulty for their infant latching-on or there were many older primiparas, which led to their difficulty in establishing breast milk. Moreover, women with GDM tended to feel tired more easily and experience emotional labiality, thus health professionals focused less on breastfeeding. Regarding the latter sub-category, respondents, for example, stated that they felt that more women with GDM preferred having a rest rather than breastfeeding.

### Barriers associated with health professionals

The barriers included in this theme consisted of the following two categories: *Issues around breastfeeding support for women with GDM at an individual level,* and *Issues around collaboration within professions and between different departments and professions.*

The first category, *Issues around breastfeeding support for women with GDM at an individual level* consisted of five subcategories. The first two were, ‘Low awareness among health professionals about GDM’ and ‘Lack of knowledge and skills among health professionals regarding GDM and breastfeeding’. For example, one respondent stated that there was not much guidance about GDM so it is essential to raise the awareness of staff and to understand the need for health guidance regarding GDM. Another example was that staff education and training about GDM was not sufficient, so health guidance for women with GDM ended up in general guidance. A third subcategory emerged about issues of support itself provided by nurses and midwives which was: ‘Lack of basic breastfeeding support for women with or without GDM’. Respondents pointed out that approaches to breastfeeding support were not actively provided from pregnancy, regardless of women’s GDM status. Moreover, the fourth subcategory was a ‘Lack of additional support for breastfeeding among women with GDM’. For example, respondents stated that GDM was not taken as a serious problem after childbirth. In particular, ‘Lack of health guidance on the effects of breastfeeding to prevent type 2 diabetes mellitus’ was the fifth subcategory identified as a barrier arising from health care providers.

*Issues around collaboration within professions and between different departments and professions,* consisted of three subcategories. First, respondents perceived ‘Differences in competence and approach to GDM and breastfeeding among nursing staff’ as a barrier. They stated that nurses and midwives have different competencies and opinions regarding breastfeeding support. This made it difficult to provide a continuity of approach when many staff are involved in care. Second, ‘Lack of coordination among different health professionals and departments’ was identified. For example, there was a lack of collaboration in breastfeeding support with NICU. Third, ‘Differing approaches among health professionals to breastfeeding for women with GDM’ was identified. Respondents stated that there were different views, about breast-feeding for women with GDM, between pediatricians and obstetricians.

### Organizational barriers

The barriers within the organization consisted of the following three categories: *Lack of resources for breastfeeding support, In-hospital support systems and policies that hinder the promotion of breastfeeding,* and *Lack of continuous interprofessional support system.*

Regarding the first category*, Lack of resources for breastfeeding support*, the two subcategories, ‘Lack of manpower’ and ‘Lack of time and opportunity to provide health guidance for breastfeeding’ were identified. For example, respondents stated that nurses and midwives were too busy to provide enough health guidance. Moreover, the third subcategory, ‘Difficulty in securing time for staff training’ was also identified. Respondents pointed out that knowledge of GDM is required, but it was difficult to hold study sessions outside working hours.

Second, the category, *In-hospital support systems and policies that hinder the promotion of breastfeeding,* was supported by three subcategories. First, ‘Hospital management systems that make breastfeeding support difficult’ was identified as a barrier. One respondent, stated that regardless if women had or did not have GDM’, it was challenging to concentrate on breastfeeding support in mixed-wards with patients having other health issues. Second, ‘Hospital policies restricting early skin to skin contact, early initiation of lactation, and rooming-in’ and third ‘Routine artificial milk supplements to infants’ were also identified as barriers. One respondent, pointed out that if the mother was using insulin the baby was routinely admitted to the GCU for 24 h, leading to mother-baby separation.

Third, *Lack of continuous interprofessional support system* was supported by two sub-categories, ‘Lack of support following discharge’ and ‘Difficulties in collaborating with other facilities’. Respondent stated that after discharge from the hospital, they followed-up with mothers at the breastfeeding outpatient clinic, but in cases where it was difficult for mothers to come to the clinic due to distance or lack of support, they were unable to offer continual support.

## Discussion

We investigated the current levels of implementation of breastfeeding support for women with GDM, which was provided in hospitals, and clarified the barriers for promoting their breastfeeding. Three main issues emerged: (1) improvements needed in basic breastfeeding support, (2) low specific breastfeeding support, and (3) numerous barriers to breastfeeding support.

### Basic breastfeeding support: current service provisions

Our research found the following forms of basic breastfeeding support, which were performed relatively well by majority of the hospitals (82.3- 95.2%): (a) *providing* general information on breastfeeding, (b) *supporting* early skin-to-skin contact followed by breastfeeding within 30 min of childbirth, (c) *guiding* in responsive breastfeeding, and (d) *providing* continuous breastfeeding support after discharge. However, of those hospitals, only about half provided such care to a great extent. Considering these results, it is unlikely that all women are provided these basic breastfeeding supports. There is strong evidence that such basic support, which includes antenatal education, early skin-to-skin contact, and responsive breastfeeding, promotes breastfeeding [18, 19]. Furthermore, these forms of support are all included in the WHO guidelines *Ten steps to successful breastfeeding* [15]. Therefore, it is necessary to initially establish support systems at the organizational level, which follow the WHO guidelines and increase the standardization of basic support for breastfeeding throughout Japan.

### Specific breastfeeding support: current service provisions for women with GDM

Regardless of the reported common challenges to breastfeeding faced by women with GDM and their infants [20–24], our findings revealed that optimal practices for women with GDM, particularly for the prevention of type 2 diabetes mellitus following GDM are not standardized in Japan. The implementation rate was considerably lower for the following specific forms of support: (a) antenatal education on handling of conditions that frequently occur after birth in women with GDM and their babies; (b) support for expressing breast milk after childbirth, when necessary; (c) avoidance of mother-infant separation and (d) education on advantages of breastfeeding in preventing type 2 diabetes mellitus. These findings indicate a lack of awareness among nurses and midwives of the need for specific support for women with GDM and thus are not educating women with GDM about the benefits of breastfeeding for the prevention of type 2 diabetes mellitus.

### Barriers to breastfeeding support: barriers to promoting breastfeeding for women with GDM as perceived by nurses and midwives

This study revealed that there are numerous barriers to providing breastfeeding support for women with GDM. First, barriers associated with mother and infant, were related to necessary *medical interventions for infant* as well as the mother’s challenges with breastfeeding. Infants born to women with GDM are at risk for hypoglycemia and may require treatment. The barrier of separate rooms for mothers and newborns has been identified in other study [25] and was also found to be a major barrier to breastfeeding support in this study.

Second, ‘Health professionals’ lack of knowledge and skills about GDM and breastfeeding’ was another barrier. A systematic review of effective support for breastfeeding [19] suggested that having trained professionals with the right knowledge and skills is fundamental. Therefore, this barrier is a significant issue to be addressed to promote breastfeeding for women with GDM. A ‘Lack of continuous interprofessional support system’ is another barrier. Although this study revealed that continuous breastfeeding consultations and phone calls following discharge are provided at a majority of hospitals throughout Japan, this service is generally a reactive support after discharge, and is not provided regularly to all breastfeeding women. McFadden et al.’s [19] systematic review found that ongoing scheduled visits that enabled breastfeeding mothers to predict the availability of support were also effective. While the McFadden et al. [19] target population in the review consisted of healthy women, their findings may arguably be applied to women with GDM in terms of a basic support. Therefore, based upon their findings, it can be reasonably inferred that optimal practice in promoting breastfeeding among women with GDM is hindered by the barriers identified in this study.

Finally, the barriers associated with health professionals and the organization, indicate the suboptimal support systems for collaboration among health professionals and for policies to promote breastfeeding for women with GDM. GDM is a complex disease that requires that glycemia be controlled as pregnancy progresses. Therefore, collaboration between and among multiple departments and professionals, such as obstetricians, pediatricians, endocrinologists, midwives, and nurses is indispensable. However, this study found that health professionals had difficulty collaborating and coordinating within their own profession as well as with other professionals as well as having ‘Differing approaches among health professionals to breastfeeding for women with GDM’. As Hall [26] states, “Each health care profession has a different culture, including values, beliefs, attitudes, customs and behaviors” (p. 188). Indeed, it is because each profession has its own specialty and perspective, that interprofessional collaboration can provide high quality patient-centered care [27]. However, given that one barrier identified in this study is the ‘Lack of collaboration among different health professionals and departments’, it is clear that those differences hinder the team support of women with GDM. Furthermore, this study reveals that hospitals have policies or protocols for women with GDM and their infants, such as routine artificial milk supplements to infants, or causing mother-baby separation, that may oppose optimal breastfeeding practices thus diminishing quality of care.

### Implications for further practice and research

Based upon the above discussion, this study proposes the following solutions to address the challenges to the promotion of breastfeeding among women with GDM. First, it is important to review hospital policies and improve the system to provide basic breastfeeding support following the *Ten Steps to Successful Breastfeeding*, [15] from pregnancy to postpartum, irrespective of the presence of GDM. Further psychometric development and use of the questionnaire used in this study could be a valuable resource for hospitals to assess their level of breastfeeding support for women with GDM. Wang et al. [28] particularly noted that nurses and midwives are the main sources of knowledge about breastfeeding for women with GDM. Therefore, it is significantly important to strengthen the breastfeeding support provided by nurses and midwives.

Second, given that there are specific barriers to successful lactation faced by women with GDM, strengthening specific and tailored assistance is required to maximize their chances of initiating and sustaining breastfeeding. Studies have found that education and tailored support were effective for women with GDM [29, 30]. For example, Stuebe et al. [29] suggested the efficacy of tailored breastfeeding support, which included face-to-face antenatal sessions regarding breastfeeding and health outcomes and proactive weekly support through text messages. Their tailored support successfully improved breastfeeding intensity and duration among women with GDM. Furthermore, several studies [31, 32] suggested the efficiency and effectiveness of educational interventions based on the Health Belief Model which indicates that a health action can prevent illness. In addition, Wallenborn et al. [33] concluded that the perceived benefits of breastfeeding were a significant factor to increase breastfeeding duration among women with GDM. Thus, providing the following information during pregnancy may increase the intention of women with GDM to breastfeed: (a) the risk of type 2 diabetes mellitus following GDM, (b) the benefit of the breastfeeding for the prevention of type 2 diabetes mellitus, (c) the risk of obesity and type 2 diabetes mellitus for babies born to women with GDM and (d) the benefit to the babies in preventing obesity and type 2 diabetes mellitus.

While it is argued that many resources are naturally required to provide such adequate support, many studies stated that resource shortages are barriers to the enhancement in health services. Likewise, this study identified the lack of resources to provide services such as lack of manpower and time to provide generous breastfeeding support. However, inadequate support, especially in the first weeks after birth, was one of the common reasons for abandoning breastfeeding [12]. Therefore, to enhance the support system for breastfeeding at hospitals where postpartum women spend the first week is indispensable as a support for promoting breastfeeding. Furthermore, as Rollins et al. [12] pointed out, although it is a limited resource, the promotion of breastfeeding has been proven to greatly contribute to society’s economy in the long-term. Considering that the promotion of breastfeeding among women with a history of GDM can reduce the incidence of type 2 diabetes mellitus in both mothers and children, strengthening support for them is of great significance.Further research is required for developing intervention programs to address the barriers for breastfeeding among women with GDM. Furthermore, there is a need for both intervention and implementation studies to evaluate and disseminate these intervention programs.

### Study limitations and strengths

This study contains a number of potential limitations. First, the response rate was relatively low, however the extent to which bias occurred would need to be statistically examined as low response rates do not automatically generate bias [34]. Yet this could have resulted in a selection bias, leading to a failure to fully represent the current situation of breastfeeding support among women with GDM throughout Japan. Second, in terms of the method, the data were collected from nurses and midwives via a questionnaire. As Althubaiti [35] pointed out, this method has a possibility of self-report bias, which is asserted to be unreliable. Therefore, methods for objectively monitoring clinical practice for breastfeeding could have strengthened the results. Although these limitations have to be considered when interpreting the study results, we nevertheless successfully investigated the current clinical practice at 296 hospitals throughout Japan in relation to breastfeeding support among women with GDM from the antenatal to postnatal period.

## Conclusion

This study reveals the current levels of implementation of breastfeeding support for women with GDM provided in hospitals in Japan, and identified barriers to promoting breastfeeding among these women. In Japan, a large majority of hospitals (82.3 - 95.2%) provided basic breastfeeding support such as giving women general information about breastfeeding, and supporting early skin-to-skin contact. However, only around 50% of hospitals told women with GDM about the benefits of breastfeeding to prevent further problems. Furthermore, there are numerous barriers perceived by nurses and midwives that prevent the promotion of breastfeeding among women with GDM. To address these issues aimed at promoting breastfeeding among women with GDM, it is important to review hospital policies and improve the system in order to provide basic breastfeeding support and become more breastfeeding friendly. Furthermore, to successfully achieve breastfeeding, specific and tailored assistance for women with GDM should be introduced.

## Data Availability

The datasets during and/or analyzed during the current study available from the corresponding author on reasonable request.
